# Chromophobe renal cell carcinoma with prolonged response to targeted therapy: a case report

**DOI:** 10.1186/1752-1947-6-115

**Published:** 2012-04-23

**Authors:** Vasiliki Michalaki, Constantine Gennatas

**Affiliations:** 1 Oncology Clinic, Second Department of Surgery, Areteion Hospital, University of Athens, 76 V. Sofias av, 115 28, Athens, Greece

## Abstract

**Introduction:**

Chromophobe renal cell carcinoma is universally accepted as a distinct subtype of renal cell carcinoma. There are conflicting reports on prognosis, and few data on response to treatment exist. Currently, we do not have any effective treatment for the metastatic disease apart from surgical procedures. Current strategies are based on results obtained in the context of clear cell-type renal cell carcinoma. Separate trials for rare histologies seem unfeasible and are unlikely to be performed. For these cases, clinical observations are an important part for advancing therapeutic insight. In recent years, novel tyrosine kinase inhibitors have been shown to have significant clinical benefit in advanced renal cell carcinoma.

**Case presentation:**

We present the case of a 43-year-old Caucasian man with advanced chromophobe renal cell carcinoma treated with the tyrosine kinase inhibitor sunitinib and subsequently with sorafenib and the mammalian target of the rapamycin inhibitor everolimus, achieving a prolonged response and significant clinical benefit. We report an unexpectedly high efficacy of everolimus as a third-line treatment in a patient with metastatic chromophobe renal cell carcinoma.

**Conclusions:**

Up to now, no published data from randomized clinical studies have addressed the question of efficacy of everolimus as a third-line treatment after failure of tyrosine kinase inhibitors. To the best of our knowledge, this case is the first report of chromophobe renal cell carcinoma treated successfully with sequential tyrosine kinase and mammalian target of rapamycin inhibitor therapy. Notably, the time on treatment with sunitinib exceeded four years. The case presented here implies that everolimus could be a viable option for patients with metastatic chromophobe renal cell carcinoma.

## Introduction

Renal cell carcinoma (RCC) accounts for approximately 1% of all cancers, and the incidence of kidney cancer, unlike other genitourinary malignancies, is rapidly increasing at 2.5% per year. Chromophobe RCC (ChRCC) represents 5% of neoplasms of renal tubular epithelium. ChRCC is a distinct type of renal cancer, is presumably derived from the intercalated cells of the collecting duct system, and exhibits a better prognosis than other types of RCC.

An enhanced understanding of the underlying biology of RCC has led to systemic therapy targeted at the vascular endothelial growth factor (VEGF) pathway as well as the the mammalian target of the rapamycin (mTOR) pathway. Agents blocking these pathway elements have demonstrated robust efficacy, offer new strategic options for patients with metastatic RCC, and have largely replaced cytokines as the standard of care in this disease.

Trials with small-molecule VEGF and platelet-derived growth factor (PDGF) receptor inhibitors, such as sunitinib and sorafenib, have shown significant clinical activity in randomized trials in advanced clear cell RCC (CCRCC). Both drugs have been approved by the US Food and Drug Administration for the treatment of metastatic RCC. Data regarding the activity of sunitinib and sorafenib in advanced ChRCC are lacking because recent trials were restricted mostly to patients with CCRCC.

In this report, we describe the case of a patient who had ChRCC and who experienced improvement in his general condition and stable disease on treatment with everolimus after a prolonged response to sunitinib treatment. This case report suggests that everolimus is an effective and appropriate treatment for this RCC tumor subtype.

## Case presentation

A 43-year-old Caucasian man presented with painless macroscopic hematuria of sudden onset and gradual deterioration. Ultrasound of his kidneys demonstrated a mass lesion with abnormal echogenicity and vascularization in the upper pole of his right kidney.

A computed tomography scan of the retroperitoneal space showed a malignant space-occupying mass in the upper pole of his right kidney. He underwent right radical nephrectomy in 2003. The nephrectomy specimen showed a 20 × 12 × 8 cm tumor at the upper pole with a homogenous, solid light brown cut surface. The tumor was limited to his kidney. The sections stained by hematoxylin and eosin showed sheets of polygonal cells with abundant granular eosinophilic cytoplasm with oval nuclei, convoluted nuclear membranes, and perinuclear cytoplasmic vacuolization. Histology revealed ChRCC.

**Figure 1:**
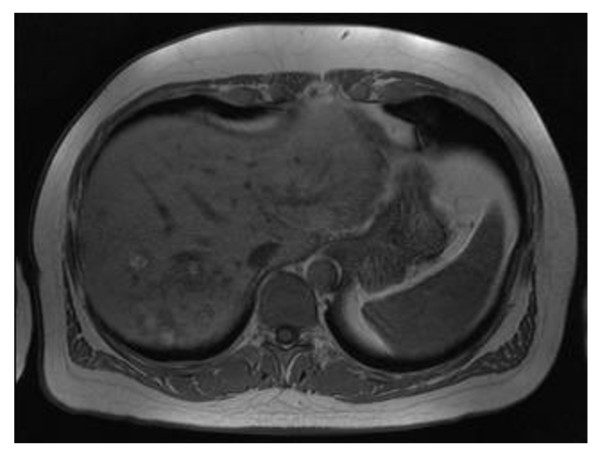
**An abdominal scan shows recurrence in the liver (multiple lesions)**.

**Figure 2:**
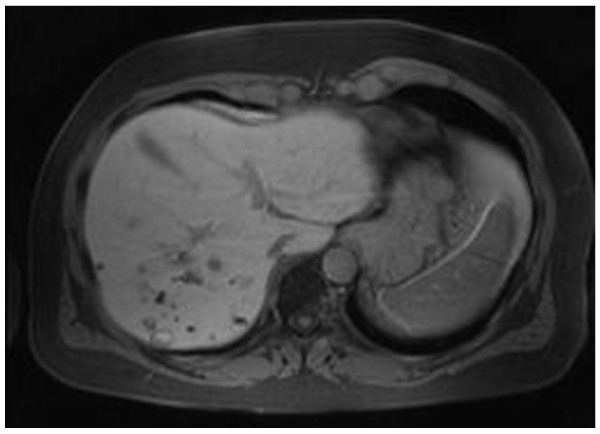
**A partial response to treatment with sunitinib**.

**Figure 3:**
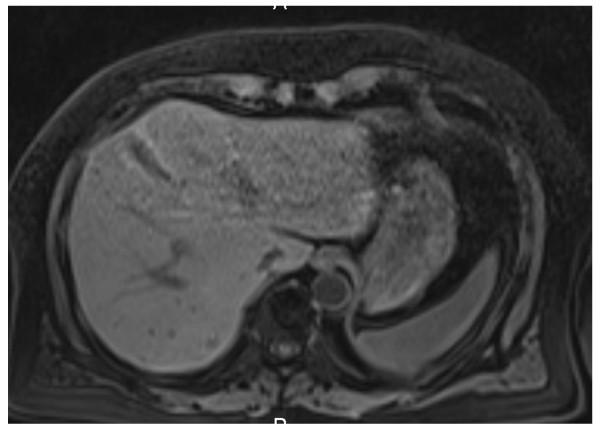
**Incremental reduction in liver disease volume on treatment with everolimus**.

Three years after nephrectomy, a routine abdominal scan showed a recurrence in his liver (Figure 1) and in bones in the chest area. The disease was considered advanced, and sunitinib 50 mg once daily on the 4/2 schedule (four weeks on treatment and two weeks off treatment cycle) was commenced. He also started zoledronic acid infusions and was treated with CyberKnife^® ^for bone metastases. A dose reduction to 37.5 mg after three months for gastrointestinal adverse effects was necessary. Our patient had a partial response to treatment and had a maximum response at nine months (Figure 2). Sunitinib was continued for four years despite some increase in bone disease burden but was stopped at 50 months as a consequence of disease progression in his liver. Sorafenib 800 mg daily was started after cessation of sunitinib for progressive disease. Follow-up imaging demonstrated stable disease. However, the next follow-up, three months later, showed progressive disease in his liver and bones. Everolimus 10 mg daily was started. At the time of this report, five months after the commencement of everolimus, there is radiologic evidence (Figure 3) of ongoing incremental reduction in liver disease volume, and our patient feels well and is tolerating treatment with no significant toxicity.

To the best of our knowledge, this case is the first report of ChRCC treated successfully with sequential tyrosine kinase and mTOR inhibitor therapy. Notably, the time on treatment with sunitinib exceeded four years.

## Discussion

RCC treatment has been classically derived from clinical trials that incorporated all histologies comprising clear cell, papillary, chromophobe, and other rarer subtypes. Most recently, novel therapies (sunitinib and sorafenib) have shown significant clinical activity in advanced RCC and have changed the standard of care in this disease [1-3]. However, pivotal studies with these drugs were performed exclusively in patients with clear cell histology. Therefore, the optimal therapy for papillary and chromophobe histologies remains unknown. Overall, ChRCC is considered to portend a good prognosis and is associated with earlier-stage tumors and longer overall survival in comparison with CCRCC. There are conflicting reports on prognosis in metastatic disease, and few data on response to treatment exist. Increased VEGF-6 and c-Kit (i.e., mast/stem cell growth factor receptor; proto-oncogene c-Kit; tyrosine-protein kinase Kit; or CD117) expressions have been reported in ChRCC, but the relevance of this to treatment is unknown [4-6]. In an expanded-access trial of sunitinib, 13% of patients had non-clear cell histology, and there was evidence of clinical activity in this group [7]. A large international study reported the outcome of 1001 patients with metastatic RCC (82 of whom had papillary histology). Five-year survival rates of papillary and clear cell subtypes were similar (about 10%). No treatment has proven to be active in papillary RCC, unlike CCRCC. One recently published series illustrated a response rate of only 5% in patients treated with these VEGF-targeted therapies [8]. Both responders received sunitinib, corresponding to an overall response rate of 17% in sunitinib-treated patients. Similarly, progression-free survival seemed to be longer in the patients treated with sunitinib in comparison with the patients treated with sorafenib (11.9 versus 5.1 months, respectively).

IIt is interesting to note that several patients who experienced progression on one anti-VEGF therapy and who were subsequently switched to another anti-VEGF therapy achieved substantial response. Although the overall duration of follow-up was relatively short, the results support the clinical rationale for continued targeting of the VEGF and c-Kit signaling pathways in non-clear cell RCC [9,10]. A phase III trial of temsirolimus also included patients with non-clear cell histology, and a subgroup analysis demonstrated an improved overall survival on temsirolimus in comparison with interferon and the combination of both drugs [11]. Choueiri and colleagues [12] retrospectively reviewed the efficacies of sunitinib and sorafenib in patients with metastatic papillary RCCs and ChRCCs. Seven patients with ChRCC were treated with sunitinib, and five were treated with sorafenib. A partial response occurred in three patients (25%), and the median progression-free survival time for patients with ChRCC was 10.6 months [12]. In summary, these data suggest that sunitinib and sorafenib have some activity in ChRCC. To the best of our knowledge, there are no published reports of the use of the mTOR inhibitors in this setting.

## Conclusions

In this report, we describe the case of a patient who had ChRCC and who experienced improvement in his general condition and stable disease on treatment with everolimus after a prolonged response to sunitinib treatment. This case report suggests that anti-VEGF agents such as sunitinib and mTOR-targeted agents such as everolimus are effective and appropriate treatments for this RCC tumor subtype.

Now that agents directed against these pathways have largely replaced immunotherapy as the standard of care, new questions have emerged and are the subject of ongoing clinical trials. Additional prospective studies and clinical experience with this rare histologic non-clear cell subtype are needed.

## Abbreviations

CCRCC: clear cell renal cell carcinoma; ChRCC: chromophobe renal cell carcinoma; mTOR: mammalian target of rapamycin; RCC: renal cell carcinoma; VEGF: vascular endothelial growth factor.

## Consent

Written informed consent was obtained from the patient for publication of this manuscript and accompanying images. A copy of the written consent is available for review by the Editor-in-Chief of this journal.

## Competing interests

The authors declare that they have no competing interests.

## Authors' contributions

CG analyzed and interpreted the patient data. VM was a major contributor in writing the manuscript. Both authors read and approved the final manuscript.
